# AACI is now an open access journal

**DOI:** 10.1186/1710-1492-5-1

**Published:** 2009-10-22

**Authors:** Richard Warrington

**Affiliations:** 1 Allergy and Clinical Immunology, University of Manitoba, MB, Canada

## Abstract

It is our pleasure to welcome you to the new website of Allergy, Asthma & Clinical Immunology (AACI).

I would like to welcome you to *Allergy, Asthma & Clinical Immunology (AACI)*, as an open access journal published by BioMed Central, the pioneering open access publisher.

*AACI *is the official Journal of the Canadian Society of Allergy & Clinical Immunology (CSACI), one of the oldest medical specialty societies in Canada and dedicated to the advancement of the knowledge and practice of allergy, clinical immunology, and asthma, and the provision of optimal patient care. AACI will publish both basic science manuscripts in allergy & immunology, as well as clinical research and review articles in these areas. We welcome manuscripts from anywhere in the World, for peer review.

Our journal was founded in 1996, as the *Canadian Journal of Allergy and Clinical Immunology*, and in 2004 became *Allergy, Asthma & Clinical Immunology*. We, the Editors-in Chief of *AACI *and the Board of Directors of CSACI, have decided that our journal should become an open-access journal because we believe that medical knowledge should be freely available to all, not just those with access to libraries or the financial ability to subscribe to medical journals. We accept that "Only presidents, editors and people with tapeworms have the right to use the editorial we."- Mark Twain

We would ask all authors to remember the words of Francis Crick, that "There is no form of prose more difficult to understand and more tedious to read, than the average scientific paper." We appreciate your efforts not to make this true of your manuscripts, since "No passion in the world is equal to the passion to alter someone else's draft."- H.G. Wells

In reviewing the available journals for publication in this field, most still require subscription, and so we hope that our open-access format will be attractive to those authors who wish to have their work available to the greatest number of researchers, clinicians, and others, wherever they are, if they have access to the Internet. We are excited about the future success of AACI, given the many benefits the open access format will afford our journal.

